# Federico di Montefeltro's hyperkyphosis: a visual-historical case report

**DOI:** 10.1186/1752-1947-2-11

**Published:** 2008-01-21

**Authors:** Anthony V D'Antoni, Stephanie L Terzulli

**Affiliations:** 1 Department of Anatomy, Touro College of Osteopathic Medicine, 230 West 125th Street, New York, NY 10027, USA; 2 Department of Clinical Immunology, Memorial Sloan-Kettering Cancer Center, 1275 York Avenue, New York, NY 10021, USA

## Abstract

**Introduction:**

The literature contains several publications describing the use of visual arts to develop observational skills in medical students. Portraits of individuals of the Italian Renaissance can be used to enhance these skills and stimulate the development of differential diagnoses in medical students. The Duke of Urbino, Federico di Montefeltro (1422–1482), lost his right eye and nasal bridge during a jousting accident in 1450. Consequently, almost every profile of him in existence today depicts his face in a left lateral view. Although some authors have described the Duke's missing nasal bridge, none have described his prominent thoracic hyperkyphosis, which is clearly discernible in two paintings by Piero della Francesca. The purpose of this report is to describe the Duke's hyperkyphosis, develop relevant differential diagnoses, and suggest a possible etiology of the convexity.

**Case presentation:**

We have examined two paintings of the Duke by Piero della Francesca – the diptych, *The Duke and Duchess of Urbino *(1465), and the *Madonna of the Egg *(1472). A MEDLINE search revealed 2 articles that were relevant to this study. This search was complemented by a search of the collection at the library of Seton Hall University, and the first author's experience studying at the University of Urbino. The historical data obtained from these searches were incorporated with the visual analysis to formulate a plausible etiology of the Duke's thoracic hyperkyphosis.

**Conclusion:**

Differential diagnoses of the Duke's thoracic hyperkyphosis include Scheuermann disease, osteoporosis, and trauma-related spinal changes. Based on the available evidence, the Duke's thoracic hyperkyphosis could have been caused by repetitive trauma to the spine due to numerous hours on horseback with heavy armor. The role that osteoporosis played in the development of the hyperkyphosis is unclear, as is whether the Duke had the convexity during childhood. The hyperkyphosis as a stylistic variant by Piero della Francesca is unlikely. This report is an example of a teaching strategy that can be used to enhance the observational skills of medical students in evidence-based medical education.

## Introduction

There are several publications in the medical literature that describe how the visual arts can be used to teach medical students clinical observational skills [1-4]. Physicians use observational skills during the history and physical examination of patients. The information gained from these observations enable physicians to identify subsets of the population with high prevalence of disease, which increases the predictive value of the tests used to confirm the diagnosis. Developing observational skills in medical students should be introduced early in medical education since it is an important component of evidence-based medicine.

Federico di Montefeltro (1422–1482), a renowned historical figure of the Italian Renaissance, was the Duke of Urbino, a small town in northeast Italy located in the mountainous region called the Apennines. Federico was a professional soldier for hire – an occupation at which he excelled and was quite prosperous – as well as a statesman and connoisseur of the humanities ("mecenate" in Italian). In 1450, he lost his right eye and bridge of his nose during a jousting accident [5], and consequently, almost every profile of him in existence today depicts him in a left lateral view so that the right side of his face cannot be observed.

We have examined two paintings of the Duke by Piero della Francesca – the diptych, *The Duke and Duchess of Urbino *(1465) at the Uffizi Gallery in Florence, Italy, and the *Madonna of the Egg *(1472) at the Pinacoteca of the Brera Academy in Milan, Italy. Although several authors have described the Duke's missing nasal bridge [5-7], none have described his prominent thoracic hyperkyphosis, which is clearly discernible in both paintings.

A MEDLINE search for English language articles published between 1960 and 2006 was performed using the non-MeSH term *Montefeltro*. A total of five articles were retrieved, and two were retained for this study (references [5] and [7]) because of their applicability to the subject. The MEDLINE search was complemented by a traditional literature search of the collection at the library of Seton Hall University (South Orange, NJ, USA), as well as the first author's experience studying at the University of Urbino in the summer of 1998. Finally, a visual analysis of the paintings was performed, and this information was incorporated with the historical data obtained from the literature searches.

## Case presentation

**Figure 1:**
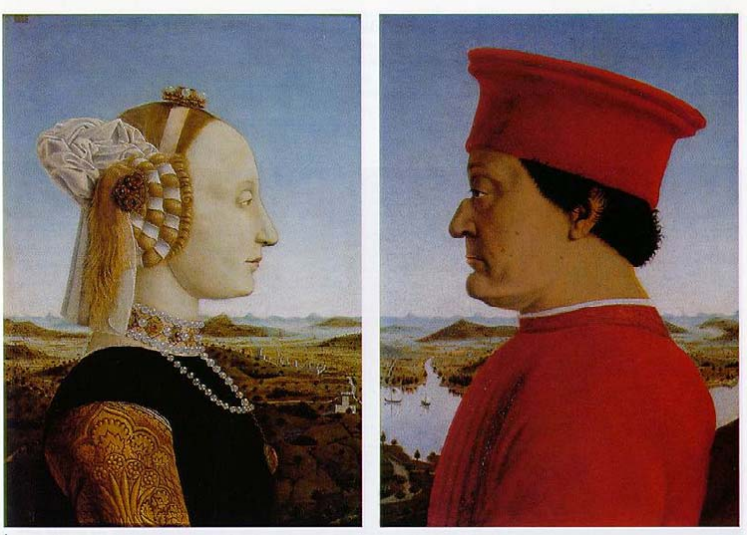
***The Duke and Duchess of Urbino *(1465) by Piero della Francesca. Uffizi Gallery, Florence, Italy**. The left panel is a portrait of Battista Sforza in a right lateral view. She faces her husband, Federico di Montefeltro, who is depicted in a left lateral view. The Duke was 43 years old when this diptych was painted. Notice the missing nasal bridge [5], moles on the cheek, and the skin folds on his jaw due to a facial skin disease that he had as a young man [13]. In addition, observe his prominent thoracic hyperkyphosis with the apex of the convexity roughly at the level of the T6–7 vertebrae. Battista, in contrast, does not have this anomaly. (Reproduced with permission from the Uffizi Gallery, Florence.)

In *The Duke and Duchess of Urbino*, the 43-year-old Duke is depicted in a left lateral view facing his wife, Battista Sforza, who is opposite him and depicted in a right lateral view (Figure 1). The head, neck, and thorax of both figures can be clearly observed in the painting. Close inspection of the Duke reveals a prominent hyperkyphosis of the thoracic spine with the apex of the convexity at approximately the level of the T6–7 vertebrae. Interestingly, there is no such deformity present in his wife. The panels of the diptych were intended to hang side by side so that the landscape in the background is contiguous.

**Figure 2:**
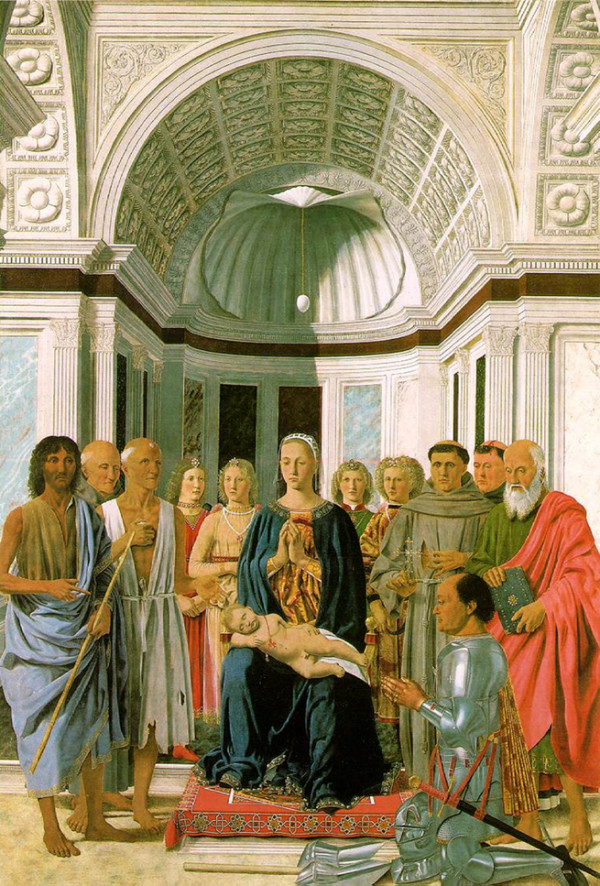
***Madonna of the Egg *(1472) by Piero della Francesca. Pinacoteca of the Brera Academy, Milan, Italy**. In this painting, the 50-year-old Duke is genuflecting and is again depicted in a left lateral view. Observation of the neck moving posteroinferiorly demonstrates the transition between skin and armor. The contour of the silver-colored armor is convex and quite striking, especially when viewed against the red cloak of the figure behind the Duke. What confounds this area is the Duke's red and gold damask cape that hangs down his back. However, the intersection of the armor and the cape reveals the prominent inferior bend of the thoracic hyperkyphosis, which is present at the same vertebral levels as in Figure 1. Apparently, his custom-made armor was crafted to accommodate his hyperkyphosis. (Reproduced with permission from the Pinacoteca of the Brera Academy, Milan.)

In the *Madonna of the Egg*, the 50-year-old Duke is shown genuflecting in the right lower quadrant of the painting, and again, he is depicted in a left lateral view (Figure 2). Here the Duke is wearing a suit of armor and his thoracic hyperkyphosis is again present at the same vertebral levels as in the previous painting. It seems as though his custom-made armor was crafted to accommodate this hyperkyphosis.

Based upon this visual analysis, the convexity portrayed in these paintings could be attributed to Scheuermann disease, osteoporosis, or trauma-related spinal changes. A stylistic variant technique by Piero della Francesca is also possible. Because the paintings were created by the same artist and depict the Duke's hyperkyphosis in both plain clothes and armor, a stylistic variant seems unlikely. A comparison of the thoracic spines of the Duke and his wife in Figure 1 reveals, quite convincingly, that the Duke had the convexity but his wife did not.

Other representations of the Duke by different artists in which he is depicted in perfect lateral view also reveal the hyperkyphosis. For example, the medal by Sperandio of Mantua depicts the Duke in left lateral view and clearly illustrates the convexity at the same vertebral level as that portrayed in the paintings by Piero della Francesca. The medal by Pauli de Ragusio (National Museum of Ireland, Dublin) also depicts the Duke in left lateral view with the convexity visible [7]. Close inspection of the Duke's neck in this medal reveals a posteroinferior slope at 45° that is interrupted by a bump, which represents the superior bend of the hyperkyphosis. A portrait of the Duke by an unknown painter, displayed in the Museo Civico di Urbania (Urbino, Italy), also reveals the Duke's convexity in left lateral view [5]. The portrait of the Duke with his son Guidobaldo by Pedro Berruguete, displayed in the Galleria Nazionale delle Marche di Urbino (Urbino, Italy), does not depict the Duke in perfect lateral view. Instead, the Duke is seen at a slightly oblique angle so that his left shoulder obscures the back, making examination of the hyperkyphosis difficult. However, the convexity is suggested because the contour of the neck slopes posteroinferiorly like that depicted in Ragusio's medal. Finally, in the painting by Joos van Ghent, the *Communion of the Apostles *(Galleria Nazionale delle Marche di Urbino), the Duke is seen on the right in the background. Again, his shoulders are oblique and the cloth draped over his left shoulder hides the hyperkyphosis. However, his face is shown in left lateral view and the slope of his posterior neck line is similar to that seen in other paintings.

Scheuermann disease is characterized by a hyperkyphosis of the thoracic spine that most often occurs in children aged 13 to 16. All existing portraits of the Duke depict him in adulthood so a comparison of his thoracic spine in childhood is not possible. Therefore, one cannot rule out the possibility of a persistent hyperkyphosis that began in childhood as a result of Scheuermann disease.

The palace of Urbino contained enough stables to accommodate 300 horses [8] and horses were the main mode of transportation at the time. As previously mentioned, war was the Duke's professional occupation and he was first sent to battle at the age of 15. Thus, it is plausible to assume that the Duke spent many hours on horseback, with and without armor, throughout his lifetime. The armor was heavy and the plates protecting the thorax were suspended by shoulder straps. This weight could certainly have influenced the curvature of the spine.

Recently, Orloff and Rapp [9] studied the effects of a 9-kg backpack on spinal curvature in healthy subjects and found significant changes in curvature as they fatigued. Other researchers have also found significant changes in posture and spinal curvature as a result of wearing backpacks [10,11]. The axial loads transmitted through the Duke's spine while on horseback [12], coupled with the weight of armor, could possibly have resulted in compression fractures of the vertebral bodies, resulting in the development of the hyperkyphosis. By the time he was an adult, the Duke's marked hyperkyphosis was present as illustrated in Figures 1 and 2. Whether the Duke had osteoporosis is unknown, but this could also be responsible for the hyperkyphosis.

## Conclusion

Based on the available evidence, the Duke's thoracic hyperkyphosis as depicted in two paintings by Piero della Francesca was probably not a stylistic variant by the artist. Possible etiologies include osteoporosis and repetitive trauma to the spine due to numerous hours on horseback with heavy armor. Whether the Duke had the anomaly during childhood is unclear.

This report illustrates how the visual arts can be used to facilitate the development of observation, an important skill for future physicians. These paintings could be presented to medical students to enhance their observational skills in an evidence-based medical education program.

## Competing interests

The author(s) declare that they have no competing interests.

## Authors' contributions

AVD designed the conceptual framework of the study and performed the literature searches. SLT was involved in the organization and preparation of the manuscript. Both authors read and approved the final manuscript.

## References

[B1] Bardes CL Gillers D Herman AE Learning to look: developing clinical observational skills at an art museum Med Educ 2001 35 12 1157 1161 10.1046/j.1365-2923.2001.01088.x 11895244

[B2] Boisaubin EV Winkler MG Seeing patients and life contexts: the visual arts in medical education Am J Med Sci 2000 319 5 292 296 10.1097/00000441-200005000-00005 10830552

[B3] Dolev JC Friedlaender LK Braverman IM Use of fine art to enhance visual diagnostic skills Jama 2001 286 9 1020 10.1001/jama.286.9.1020 11559280

[B4] Lazarus PA Rosslyn FM The arts in medicine: setting up and evaluating a new special study module at Leicester Warwick Medical School Med Educ 2003 37 6 553 559 10.1046/j.1365-2923.2003.01537.x 12787379

[B5] Santoni-Rugiu P Massei A The legend and the truth about the nose of Federico, Duke of Urbino Br J Plast Surg 1982 35 3 251 257 6758899

[B6] de La Sizeranne R Federico di Montefeltro: capitano, principe, mecenate (1422-1482) 1972 Urbino , Argalià Editore

[B7] Winters HPJ Federico da Montefeltro, duke of Urbino (1422-1482): the story of his missing nasal bridge Br J Plast Surg 1982 35 3 247 250 6758898 10.1016/s0007-1226(82)90109-6

[B8] Prescott O Princes of the renaissance 1969 New York , Random House

[B9] Orloff HA Rapp CM The effects of load carriage on spinal curvature and posture Spine 2004 29 12 1325 1329 10.1097/01.BRS.0000127185.74191.B1 15187633

[B10] Korovessis P Koureas G Papazisis Z Correlation between backpack weight and way of carrying, sagittal and frontal spinal curvatures, athletic activity, and dorsal and low back pain in schoolchildren and adolescents J Spinal Disord Tech 2004 17 1 33 40 10.1097/00024720-200402000-00008 14734974

[B11] Korovessis P Koureas G Zacharatos S Papazisis Z Backpacks, back pain, sagittal spinal curves and trunk alignment in adolescents: a logistic and multinomial logistic analysis Spine 2005 30 2 247 255 10.1097/01.brs.0000150505.59690.1b 15644765

[B12] Palanichamy MS Patil MK Ghista DN Minimization of the vertical vibrations sustained by a tractor operator, by provision of a standard-type tractor seat suspension Ann Biomed Eng 1978 6 2 138 153 10.1007/BF02584540 717865

[B13] Lightbown R Piero della Francesca 1992 New York , Abbeville Press

